# Combat injury, pain, and mental health outcomes in US Army service members

**DOI:** 10.1017/S0033291726103584

**Published:** 2026-03-24

**Authors:** Marcus G. Wild, Laura Campbell-Sills, Xiaoying Sun, Ronald C. Kessler, David M. Benedek, Robert J. Ursano, Sonia Jain, Murray B. Stein

**Affiliations:** 1 VISN 17 COE: Veterans Integrated Services Network 17 Center of Excellence for Research on Returning War Veterans, Waco, USA; 2 Central Texas Veterans Health Care System, Temple, USA; 3 Department of Psychiatry, University of California San Diego, La Jolla, USA; 4 Herbert Wertheim School of Public Health and Human Longevity Science, University of California San Diego, La Jolla, USA; 5 Department of Health Care Policy, Harvard Medical School, Boston, USA; 6 Department of Psychiatry, Uniformed Services University of the Health Sciences, Bethesda, USA

**Keywords:** combat injury, mental health, pain, US service members, PTSD, depression, Major Depressive Episode, combat, deployment, trauma, pain interference, pain catastrophizing

## Abstract

**Background:**

The impact of combat injury on the development of chronic pain and mental health concerns in combat-exposed populations is unknown. This study examined associations of combat injury and injury–related pain with pain-related factors and mental health outcomes, and potential mediation of the relation between combat injury and mental health outcomes by pain-related factors.

**Methods:**

Pain interference, pain catastrophizing, pain intensity, post-traumatic stress disorder (PTSD), and major depressive episode (MDE) were assessed in (1) a probability sample of US Army soldiers and veterans cross-sectionally and (2) US Army soldiers before and 1, 3, and 9 months after deployment to Afghanistan. Associations among these variables were modeled using logistic regression and multiple mediation analyses.

**Results:**

Among 5003 service members with cross-sectional data, combat injury–related pain was associated with increased odds of clinically significant pain intensity (OR=2.69), pain interference (OR=3.69), MDE (OR=2.17), and PTSD (OR=3.96) relative to pain from other injuries and conditions. Among 4645 service members assessed pre- and post-deployment, combat injury was associated with increased odds of new-onset pain interference (OR=2.78), pain catastrophizing (OR=2.75), PTSD (OR=4.06), and MDE (OR=2.56) 3 months post-deployment, and PTSD (OR=2.86) and MDE (OR=1.74) 9 months post-deployment. Pain-related factors mediated the relations of combat injury with post-deployment PTSD and MDE.

**Conclusions:**

Combat injury is associated with greater odds of pain interference, pain catastrophizing, PTSD, and MDE compared to other sources of pain in a cohort of US service members. Efforts to address pain-related factors following combat injury may mitigate the risk of subsequent chronic pain and mental health disorders.

Chronic pain – pain lasting for 3 months or longer – is common and a leading cause of disability, notably among US service members and veterans (Dieleman et al., 2020; Nahin, 2017; Reed et al., 2024). Of US service members and veterans with chronic pain, over half report functional impacts – reduced ability to engage in activities of living – due to chronic pain (Barry et al., 2003; Reed et al., 2025).

Exposure to combat-related injuries may be one reason why US service members and veterans are at higher risk for chronic pain and pain-related disability than the general population. Central to the biopsychosocial model of chronic pain (Begum et al., 2025; Cook, Brawer, & Vowles, 2006; Gatchel et al., 2007) is a fear–avoidance cycle, where fear of pain maintains chronic pain (G. J. Asmundson, Norton, & Norton, 1999; Cook et al., 2006). Accordingly, combat-related injuries (e.g. gunshot wounds and explosions), which may provoke sustained fear reactions and avoidance, may increase risk for chronic pain. Evidence suggests that combat injury is related to increased odds of chronic pain (Toblin et al., 2014), higher frequency of moderate-to-severe chronic pain (Vollert et al., 2024), and higher prevalence of post-injury pain (Kumar et al., 2024) compared to noncombat injury. However, evidence remains limited on differences in intensity of pain from combat injuries compared to other sources of pain and on the psychological and functional impacts of combat injury–related pain. Investigating the link between combat injuries and psychological outcomes could inform both theory on fear as a mechanism of chronic pain development and treatment of combat injury in ongoing global conflicts.

Specific psychological outcomes relevant to those with chronic pain include post-traumatic stress disorder (PTSD) and major depressive disorder (MDD). PTSD and chronic pain are highly comorbid due to shared precipitating events, such as traumatic injury, and a mutual maintenance of symptoms from avoidance of feared stimuli (A. J. N. Asmundson et al., 2025; Liedl et al., 2010; Norman, Stein, Dimsdale, & Hoyt, 2008; Reed et al., 2024; Sharp & Harvey, 2001). Combat injury may play an outsized role in the comorbidity between PTSD and chronic pain, given its occurrence within a fear-inducing context (Afari et al., 2009; Vollert et al., 2024). MDD is also closely connected to chronic pain (Aaron et al., 2025; Dudeney et al., 2024) and frequently comorbid with PTSD (Androulakis et al., 2021; Boska, Bishop, & Ashrafioun, 2021). Yet it remains unknown whether combat injury is directly associated with an increased risk of these mental health conditions.

In addition to psychological sequelae, chronic pain following traumatic injuries affects psychosocial functioning, such as the ability to work or engage in social activities (Gironda et al., 2009). Pain-related interference in psychosocial functioning (i.e. pain interference) is therefore the primary target of pain-management interventions (Krebs et al., 2009; Ord et al., 2021). Furthermore, psychological features of chronic pain are commonly targeted by interventions. Pain catastrophizing – extreme concerns about pain and its consequences – is a target of evidence-based interventions for chronic pain and associated with psychosocial functioning in veterans with pain (Murphy, Cordova, & Dedert, 2022). Pain interference and pain catastrophizing are also relevant to the fear–avoidance model of chronic pain, whereby catastrophizing about future pain leads to increased fear of activity and subsequent functional interference. It is paramount to better understand how combat injury may relate to these critical characteristics of pain in order to improve care.

Finally, it is important to understand whether the attribution of pain to a combat injury is associated with different pain characteristics than the attribution of pain to another source (e.g. a noncombat training injury, medical condition, etc.). In addition to the unique impacts of fear that a combat injury may cause on pain development, pain attributions – what is thought to be the cause(s) of pain – are also known to differentially predict functioning and treatment (e.g. Ashar et al., 2023); however, no work at present has examined how a person’s attribution of their pain to a combat injury might associate with mental health outcomes. This gap could be particularly relevant to service members and veterans, who may be exposed to a number of potential sources of pain.

There are several clinical and theoretical implications from a better understanding of combat injury as a source of pain. Clinically, if combat injury is associated with greater pain catastrophizing and pain interference, triaging of service members who are injured in combat to early intervention that emphasizes coping strategies to reduce pain catastrophizing (e.g. cognitive behavioral therapy for chronic pain) and increased focus on early mobility following injury could improve outcomes. Additionally, because of the role of fear as a perpetuating mechanism in the mutual maintenance of pain and PTSD, early treatment of combat injury-related pain may mitigate the risk of PTSD subsequent to a combat injury by reducing vigilance and trauma-related attributions of painful stimuli as threats. Furthermore, the chronic stress resulting from unresolved pain catastrophizing and pain interference could have negative impacts on mood (e.g. MDD) that could be prevented by early intervention. However, these points are currently speculative due to the lack of existing evidence on the link between combat injury–related pain and mental health outcomes.

Given these gaps in knowledge, we first cross-sectionally examined the difference in odds of pain interference and adverse mental health outcomes associated with combat injury–related pain compared to noncombat injury–related pain in a probability sample of current and former US Army soldiers. Next, we longitudinally evaluated (1) the association of combat injury with subsequent pain interference, pain catastrophizing, and mental health outcomes, and (2) the mediation of the relation between combat injury and mental health outcomes by pain interference, pain catastrophizing, and clinically significant pain intensity using data from three US Army combat brigades surveyed before and at several time points following a combat deployment.

## Methods

### Participants and procedures

Data were from the Army Study to Assess Risk and Resilience in Servicemembers (Army STARRS) and the STARRS-Longitudinal Study (STARRS-LS). The design and procedures of Army STARRS and STARRS-LS are detailed elsewhere (Kessler et al., 2013a; Ursano et al., 2014). Briefly, STARRS-LS recruited a probability sample of service members who completed baseline surveys while on active duty as part of the Army STARRS New Soldier Study (NSS), All Army Study, or Pre/Post Deployment Study (PPDS; Kessler et al., 2013a; Ursano et al., 2014) and agreed to their survey responses being linked to their Army/Department of Defense (DoD) administrative data.

Cross-sectional analyses for the current study used data from wave 1 of STARRS-LS (LS1). A total of 14,508 current and former US Army soldiers participated in LS1 between September 2016 and April 2018. The LS1 analysis sample was restricted to participants whose baseline data were collected in the NSS (*n* = 6333), representing a cohort who enlisted between April 2011 and November 2012 and whose LS1 data were collected ~6 years post-enlistment. The LS1 sample was further constrained to those who reported pain (*n* = 5003), to enable comparisons of combat injury–related pain to pain from other sources.

Longitudinal analyses used data from the PPDS (January 2012–April 2014), which involved a baseline assessment before deployment to Afghanistan in 2012(T0), a brief assessment within ~1 month of returning(T1), and subsequent follow-ups 3 months(T2) and 9 months(T3) post-deployment. PPDS methods are detailed elsewhere (Campbell-Sills et al., 2023; Kessler et al., 2013a, 2013b; Ursano et al., 2014). Briefly, 9949 service members were on duty in the 3 brigade combat teams (BCTs) eligible for recruitment, and 9488 (95.3%) consented to participate. Of those who consented, 8558 (86.0%) provided complete data and permitted Army/DoD record linkage. Because the analysis used data from all waves, the sample was restricted to the 60.0% of deployed service members with data at all time points (*n* = 4645). All participants provided informed consent, and study procedures were approved by the Institutional Review Boards of the collaborating institutions.

### Measures

#### *Combat injury*–*related pain and combat injury*

Combat injury–related pain was defined in the LS1 survey by an item assessing the primary source of pain, for which ‘A combat injury’ was an option. Comparisons in LS1 were made between those who endorsed combat injury as their primary source of pain (1) and those with pain who did not endorse a combat injury as the primary source of that pain (0). In PPDS, combat injury was defined as responding ‘1 time’ or greater to the T1 survey item that queried being wounded during the index deployment. Comparisons were made between those who endorsed being wounded (1) and those who did not endorse being wounded (0).

##### Pain intensity, pain interference, and pain catastrophizing

Pain intensity was assessed in both LS1 and PPDS with the numeric rating scale (NRS; average pain from [0] *No pain* to [10] *Pain as bad as could be*). The NRS is reliable for the assessment of pain intensity (Ferreira-Valente, Pais-Ribeiro, & Jensen, 2011; Jensen, Chen, & Brugger, 2003). Clinically significant pain intensity (≥4 on the NRS) was defined per established guidelines (Jones, Vojir, Hutt, & Fink, 2007).

Pain interference was assessed in the LS1 survey by the item, ‘How much did pain interfere with your normal work (including work outside the home and housework) in the past 30 days?’ with a five-point response scale ranging from (0) *Not at all* to (4) *Extremely.* Due to right-skewed distributions, responses were dichotomized, with pain interference defined as (2) *Moderately* or greater. At PPDS T0 and T2, pain interference was assessed with two items from the Graded Chronic Pain Scale (Von Korff, Ormel, Keefe, & Dworkin, 1992) assessing interference with work and social activities on a scale from (0) *None of the time* to (4) *All or almost all the time.* Respondents were coded as having pain interference if they endorsed (2) *Some of the time* or greater on either item. Internal consistency of the dichotomized items was high (*α* = 0.86). At T3, the pain interference assessment was restricted to interference with work, with pain interference coded as present if the item was rated (2) *Some of the time* or greater.

Pain catastrophizing was also assessed in PPDS, through three items from the Pain Catastrophizing Scale (Sullivan, Bishop, & Pivik, 1995) rated from (0) *None of the time* to (4) *All or almost all the time.* These same items were assessed at T0 and T2. Pain catastrophizing was coded as present if any of these three items were rated (2) *Some of the time* or greater, with the overall score demonstrating acceptable internal consistency (*α* = 0.78).

##### Mental health

PTSD was assessed via adapted versions of the PTSD Checklist-Civilian Version (NSS and PPDS T0 surveys) and PTSD Checklist for DSM-5 (PCL-5; LS1 and PPDS T2 and T3 surveys; Blevins et al., 2015; Weathers et al., 1993). Major depressive episodes (MDEs) were evaluated in all surveys using items adapted from the Composite International Diagnostic Interview Screening Scales (CIDI-SC; Kessler & Üstün, 2004). Questions were asked about the experience of mental health symptoms over the respondent’s lifetime in the NSS and PPDS T0 surveys and over the past 30 days in the PPDS T2 and T3 and LS1 surveys. The outcomes used for this study were binary variables of (1) meets probable diagnosis or (0) does not meet probable diagnosis. Army STARRS survey-based diagnoses demonstrated good agreement with diagnoses based on clinical interviews in a previous study (Kessler et al., 2013a, 2013b).

##### Covariates

Covariates in each model included baseline sociodemographic features (age, sex, race, and ethnicity). Raw race and ethnicity responses were categorized as Black, Hispanic, Other, and Non-Hispanic White. Baseline lifetime diagnosis of PTSD and MDE were also included in all models to account for pre-enlistment (the NSS baseline for the LS1 sample) or pre-deployment (PPDS T0) status on the outcome of interest. In the LS1 analysis, the models also adjusted for current military status (active duty, activated Guard/Reserve, deactivated Guard/Reserve, separated, or retired) and history of combat deployment (yes or no). In PPDS, the models also adjusted for BCT, number of previous combat deployments (0, 1, or > 1), and number of deployment stressors (minus the focal exposure of being wounded; Stein et al., 2015).

### Data analysis

Weights-adjusted values were used for sample description and statistical models. For the LS1 sample, weights included nonresponse and poststratification adjustments for Army STARRS baseline and STARRS-LS follow-up survey data, oversampling of baseline respondents with mental health concerns, and underrepresentation of difficult-to-recruit participants in STARRS-LS. The weighting procedures for LS1 have previously been described (Naifeh et al., 2023).

For the PPDS sample, weights included (1) a propensity-based adjustment for baseline attrition; (2) post-stratification based on key demographic and Army service characteristics to generalize to soldiers who deployed to Afghanistan after T0; and (3) a propensity-based attrition adjustment to account for incomplete data in one or more of the three follow-up waves. More information about the weighting of Army STARRS data is available elsewhere (Kessler et al., 2013b).

For the PPDS sample, we assessed the association of combat injury at T1 with the new onset of pain interference and catastrophizing at T2 and T3 using weights-adjusted logistic regression models.

For both LS1 and PPDS samples, Spearman correlations were used for bivariable associations, and weights-adjusted multivariable logistic regression was conducted to assess the independent associations of combat injury–related pain (in LS1), combat injury (in PPDS), clinically significant pain intensity, pain interference, and pain catastrophizing (only in PPDS) with mental health outcomes adjusted for relevant covariates. In the LS1 sample, the interaction of combat injury-related pain and pain interference was included to explore the potential increased mental health impact of functional interference due to combat injury compared to other pain sources. Adjusted odds ratios (ORs) with 95% confidence intervals were reported. Variance inflation factors ranged between 1.10 and 2.31 across all models and terms, indicating that multicollinearity was unlikely among the variables included.

For the PPDS sample, we also examined the extent to which associations between combat injury and mental health outcomes were explained by pain variables (clinically significant pain intensity, pain interference, and pain catastrophizing) using the ‘mma’ package in R to run multiple mediation analysis (Yu & Li, 2017). The models also adjusted for the relevant covariates. The estimated total, direct, and indirect (mediation) effects on the log-odds of the outcome were reported with confidence intervals estimated using bootstrapping. All analyses were performed between November 2024 and July 2025 in R version 4.4.1 (R Core Team, 2018).

## Results

### Sample characteristics

The descriptive statistics, including raw frequencies and weights-adjusted percentages, for both samples are in Table 1. Of the 6333 NSS respondents who completed surveys at LS1, 5003 (80%) endorsed past-30-day pain ‘a little of the time’ or more. Of these respondents, 2453 (50.5%) endorsed clinically significant pain intensity, 1442(29.7%) endorsed pain interference, 1101 (22.5%) endorsed MDE, and 1180 (22.9%) endorsed PTSD. In the LS1 sample with pain, clinically significant pain intensity and pain interference were significantly, positively correlated (*ρ* = .54).

**Table 1.** Baseline descriptive statistics for the study samples

	Cross-sectional sample – LS1 (*N* = 5003)	Longitudinal sample – PPDS (*N* = 4645)
**Sex, no. (%)**		**At T0**
Female	1177 (19.7%)	276 (5.2%)
Male	3826 (80.3%)	4358 (94.8%)
**Age, mean (SE), years**	26.07 (0.07)	26.96 (0.20)
**Race/ethnicity, no. (%)**		
Hispanic/Latino/a/e	568 (12.7%)	762 (16.2%)
Non-Hispanic Black	825 (18.6%)	467 (11.2%)
Non-Hispanic, Other Race	272 (5.4%)	384 (7.6%)
Non-Hispanic White	3338 (63.3%)	3032 (65.0%)
**Prior combat deployments, no. (%)**		
0	3702 (72.9%)	2402 (44.6%)
1+	1288 (27.1%)	2228 (55.4%)
**Military status, no. (%)**		
Active duty	1169 (24.0%)	4645 (100%)
Activated national guard/reserves	297 (4.7%)	–
Deactivated guard/reserves	1567 (25.2%)	–
Retired	188 (4.3%)	–
Separated	1782 (41.8%)	–
**Pain intensity (out of 10), mean (SE)**	3.88 (0.04)	2.69 (0.98)
**Clinical pain intensity, no. (%)**		
No	2548 (49.5%)	3118 (67.3%)
Yes	2453 (50.5%)	1512 (32.7%)
**Combat injury**–**related pain/combat injury, no. (%)**		**At T1**
No	4819 (96.6%)	4362 (94.5%)
Yes	184 (3.4%)	263 (5.5%)
**Pain catastrophizing, no. (%)**		**At T2**
No	–	3756 (82.1%)
Yes	–	861 (17.9%)
**Pain interference, no. (%)**		**At T2**
No	3559 (70.3%)	2770 (60.7%)
Yes	1442 (29.7%)	1849 (39.3%)
**PTSD, no. (%)**	**Past 12-Months**	**Past 30-Day at T2**
No	3823 (77.1%)	4288 (92.3%)
Yes	1180 (22.9%)	357 (7.7%)
**MDE, no. (%)**	**Past 12-Months**	**Past 30-Day at T2**
No	3902 (77.5%)	4315 (93.3%)
Yes	1101 (22.5%)	330 (6.7%)

*Note:* Table contains raw frequencies and weights-adjusted percentages/means based on the sampling approaches taken to each study. LS1 survey data were used in the cross-sectional analysis. PPDS was a longitudinal survey comprised of a baseline (T0) assessment, and 1 (T1), 3 (T2), and 9 months (T3) following return from deployment. LS1, STARRS Longitudinal Survey-Wave 1; PPDS, pre–post-deployment survey; PTSD, post-traumatic stress disorder; MDE, major depressive episode.

In the longitudinal sample (PPDS), of the 4645 soldiers who completed all waves, 1741 (36.7%) endorsed clinically significant pain intensity at T2, 1136 (23.6%) endorsed pain interference at T2 and 1106 (18.2%) at T3, 861 (17.9%) endorsed pain catastrophizing at T2 and 599 (13.8%) at T3, 357 (7.7%) reported PTSD and 330 (6.7%) endorsed MDE at T2, and 547 (9.6%) endorsed PTSD and 314 (5.7%) endorsed MDE at T3. In this sample, clinically significant pain intensity was significantly correlated with pain interference (T2: *ρ* = .60; T3: *ρ* = .59 and pain catastrophizing (T2: *ρ* = .53; T3: *ρ* = .45) at T2 and T3.

### Combat injury–related pain in LS1

Among those endorsing pain at LS1, combat injury–related pain relative to pain from other injuries or conditions was associated with a 2.17 (95% CI: 1.44–3.27) times increase in odds of past-12-month MDE after controlling for covariates. When pain intensity and interference were added to the model, combat injury–related pain was no longer significantly associated with MDE (Table 2). Instead, pain interference (OR = 3.04, 95% CI: 2.47–3.74) and clinically significant pain intensity (OR = 2.18, 95% CI: 1.64–2.91) were associated with increased odds of MDE. There was, however, a significant interaction between combat injury–related pain and pain interference, such that those respondents with combat injury–related pain and pain interference had magnified odds of MDE compared to those with pain interference alone (OR = 5.46, 95% CI: 1.71–17.44).

**Table 2.** Weights-adjusted multivariable logistic regression of past-12-month MDE and PTSD in LS1

Predictor	OR	95% CI			*χ*^2^		p
		*LL*	*UL*				
**MDE**							
Combat injury–related pain	0.35	0.10	1.18		2.87		0.090
Pain interference	2.90	2.34	3.60		94.20		<0.0005
Clinical pain intensity	2.19	1.64	2.91		28.62		<0.0005
Age	1.00	0.98	1.03		0.12		0.732
Female	1.46	1.19	1.78		13.36		<0.0005
Non-Hispanic Black	0.80	0.62	1.04		–		–
Hispanic	0.96	0.70	1.30		–		–
Non-Hispanic other	0.90	0.55	1.50		2.91		0.406
Activated guard/reserve	0.76	0.48	1.21		–		–
Deactivated guard/reserve	1.77	1.23	2.53		–		–
Retired	5.56	3.78	8.20		–		–
Separated	3.96	2.86	5.48		138.17		<0.0005
Previously deployed	1.21	0.94	1.57		2.18		0.140
Lifetime MDE prior to LS1	14.27	10.38	19.61		268.22		<0.0005
Combat injury × pain interference	5.46	1.71	17.44		8.23		0.004
**PTSD**							
Combat injury–related pain	2.89	1.85	4.51		21.90		<0.0005
Pain interference	2.68	2.07	3.46		56.30		<0.0005
Clinical pain intensity	1.93	1.51	2.46		28.04		<0.0005
Age	0.99	0.96	1.01		1.34		0.248
Female	1.76	1.47	2.10		38.06		<0.0005
Non-Hispanic Black	0.86	0.67	1.11		–		–
Hispanic	1.17	0.86	1.59		–		–
Non-Hispanic other	0.71	0.44	1.13		5.47		0.141
Activated guard/reserve	1.01	0.69	1.48		–		–
Deactivated guard/reserve	1.55	1.17	2.06		–		–
Retired	3.29	2.12	5.12		–		–
Separated	2.36	1.79	3.10		60.02		<0.0005
Previously deployed	1.37	1.08	1.74		6.83		0.009
Lifetime PTSD prior to LS1	6.66	5.36	8.27		294.33		<0.0005

*Note*: Total *N* = 4987. OR = odds ratio; CI, confidence interval; *LL*, lower limit; *UL*, upper limit; MDE, major depressive episode; PTSD, post-traumatic stress disorder. Models with main effects only were retained when interaction terms were nonsignificant.

Combat injury–related pain relative to pain from other injuries or conditions was also associated with a 3.96 (95% CI: 2.71–5.79) times increase in odds of past-12-month PTSD. When pain intensity and interference were added to the model, combat injury–related pain remained significantly associated with PTSD (Table 2; OR = 2.89, 95% CI: 1.85–4.51). Additionally, pain interference (OR = 2.68, 95% CI: 2.07–3.46) and clinically significant pain intensity (OR = 1.93, 95% CI: 1.51–2.46) were associated with increased odds of PTSD. The interaction term was nonsignificant in this analysis, so the main-effects model was accepted as the final model.

### Combat injury in PPDS

#### New-onset pain interference and catastrophizing

Combat injury was associated with increased odds of new-onset pain interference and catastrophizing at both T2 and T3. See Supplementary Material for details.

#### PTSD

Combat injury was associated with increased odds of past-30-day PTSD at T2 (OR = 4.06, 95% CI: 2.68–6.16) compared to no combat injury. When T1 deployment stress and T2 pain variables were added to the model, combat injury remained associated with increased odds of PTSD (Table 3; OR = 1.94, 95% CI: 1.19–3.14), as were pain interference (OR = 2.54, 95% CI: 1.65–3.92) and pain catastrophizing (OR = 2.10, 95% CI: 1.43–3.07). Combat injury was again associated with past-30-day PTSD at T3 (OR = 2.86, 95% CI: 1.99–4.09). When pain variables were added to the model, the association was attenuated but still significant, with pain variables also exhibiting significant associations with past-30-day PTSD (Table 3).

**Table 3.** Weights-adjusted multivariable logistic regression of past-30-day PTSD and MDE in PPDS T2 and T3

Predictor	OR	95% CI		*χ*^2^	p	OR	95% CI		*χ*^2^	p
		*LL*	*UL*				*LL*	*UL*		
**T2**	**PTSD**					**MDE**				
Combat injury	*1.94*	*1.19*	*3.14*	*7.15*	*0.007*	1.32	0.81	2.15	1.21	0.271
Pain interference	*2.54*	*1.65*	*3.92*	*17.70*	*<0.0005*	*3.87*	*2.58*	*5.81*	*42.69*	*<0.0005*
Pain catastrophizing	*2.10*	*1.43*	*3.07*	*14.36*	*<0.0005*	*1.74*	*1.16*	*2.60*	*7.11*	*0.008*
Clinical pain intensity	1.24	0.89	1.73	1.63	0.202	1.09	0.67	1.77	0.11	0.740
Deployment stress	*1.30*	*1.24*	*1.36*	*115.64*	*<0.0005*	*1.18*	*1.12*	*1.24*	*40.19*	*<0.0005*
Age	1.02	0.99	1.04	2.22	0.136	1.02	0.99	1.05	1.28	0.258
Female	*1.74*	*1.02*	*2.98*	*4.15*	*0.042*	*1.88*	*1.14*	*3.10*	*6.06*	*0.014*
Non-Hispanic Black	0.89	0.51	1.53	–	–	1.47	0.92	2.35	–	–
Hispanic	1.37	0.97	1.94	–	–	0.83	0.56	1.22	–	–
Non-Hispanic other	1.42	0.85	2.36	4.50	0.212	1.36	0.87	2.11	6.09	0.107
Previously deployed once	1.31	0.92	1.85	–	–	0.85	0.61	1.18	–	–
Previously deployed 2+	1.01	0.69	1.49	3.44	0.179	0.70	0.48	1.01	3.59	0.166
Lifetime PTSD/MDE (pre-deployment)	*3.56*	*2.47*	*5.14*	*46.43*	*<0.0005*	*8.09*	*5.69*	*11.51*	*135.14*	*<0.0005*
**T3**	**PTSD**					**MDE**				
Combat injury	*1.72*	*1.21*	*2.46*	*9.02*	*0.003*	1.07	0.64	1.79	0.07	0.792
Pain interference	*1.66*	*1.22*	*2.27*	*10.28*	*0.001*	*1.84*	*1.35*	*2.52*	*14.67*	*<0.0005*
Pain catastrophizing	*1.74*	*1.29*	*2.34*	*13.18*	*<0.0005*	1.34	0.95	1.90	2.74	0.098
Clinical pain intensity	*1.46*	*1.09*	*1.94*	*6.51*	*0.011*	*1.80*	*1.30*	*2.49*	*12.57*	*<0.0005*
Deployment stress	*1.19*	*1.15*	*1.24*	*76.28*	*<0.0005*	*1.14*	*1.10*	*1.19*	*43.12*	*<0.0005*
Age	1.01	0.98	1.03	0.23	0.628	1.02	0.99	1.04	2.03	0.155
Female	*2.06*	*1.37*	*3.10*	*12.04*	*0.001*	*2.12*	*1.14*	*3.93*	*5.70*	*0.017*
Non-Hispanic Black	1.11	0.70	1.75	–	–	1.19	0.76	1.86	–	–
Hispanic	1.31	1.05	1.64	–	–	1.08	0.76	1.54	–	–
Non-Hispanic other	1.29	0.81	2.05	6.46	0.091	1.19	0.78	1.82	1.18	0.759
Previously deployed once	0.93	0.69	1.25	–	–	0.90	0.63	1.28	–	–
Previously deployed 2+	1.00	0.76	1.33	0.35	0.841	1.00	0.73	1.37	0.51	0.774
Lifetime PTSD/MDE (pre-deployment)	*3.87*	*2.90*	*5.16*	*85.10*	*<0.0005*	*4.30*	*3.15*	*5.88*	*84.02*	*<0.0005*

*Note*: Total *N* = 4456. OR, odds ratio; CI, confidence interval; *LL*, lower limit; *UL*, upper limit; MDE, major depressive episode. Reference categories: Female: Male; Race: non-Hispanic White; Deployments: Not previously deployed. Models also adjusted for Brigade Combat Team. Significant associations are italicized.

#### Joint mediation of the relation between combat injury and PTSD by pain variables

**Figure 1.**
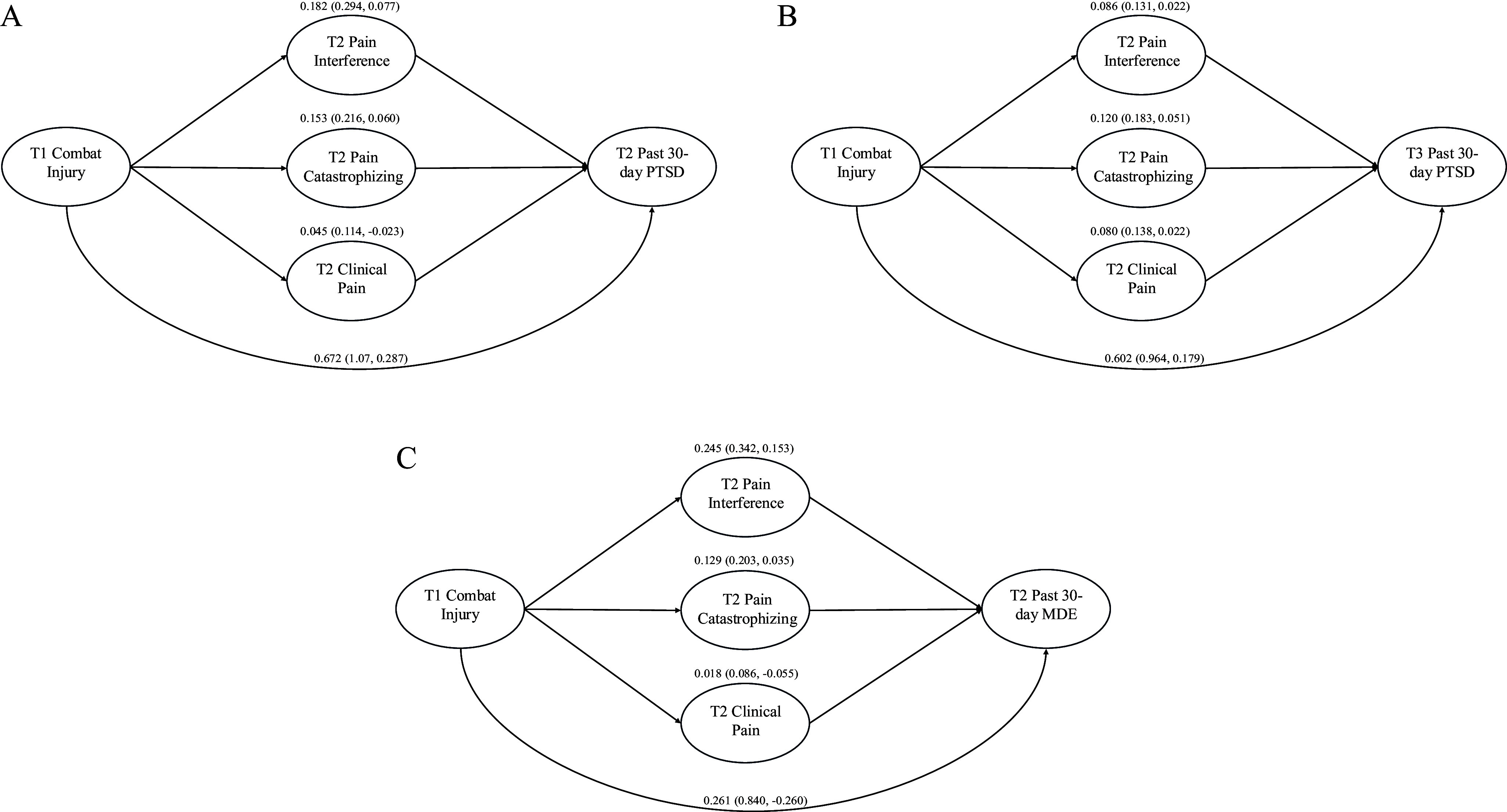
Multiple mediation models of (a) the relation between T1 combat injury and T2 past-30-day PTSD, (b) the relation between T1 combat injury and T3 past-30-day PTSD, and (c) the relation between T1 combat injury and T2 past-30-day MDE. The coefficients for the full effect of combat injury on PTSD/MDE and the mediation effect of each mediator are shown on the log odds scale.

In the multiple mediation analysis (Figure 1a), the total effect of combat injury on past-30-day PTSD at T2 indicated that those with a combat injury had 2.89 times greater odds of PTSD than those without a combat injury (log odds = 1.06, 95% CI: 0.61–1.50). The joint mediation effect (i.e. total indirect effect) indicated 34% of the increase in odds of PTSD associated with combat injury was explained by the pain variables (log odds = 0.36, 95% CI: 0.22–0.52). Most of the mediation effect was attributable to pain interference (17.2%) and pain catastrophizing (14.4%). For T3 PTSD (Figure 1b), the odds of past-30-day PTSD were 2.43 times greater for those with a combat injury than those without (log odds = 0.89, 95% CI: 0.45–1.25). The joint mediation effect indicated 33% of the increase in odds of T3 PTSD due to combat injury was explained by the pain variables (log odds = 0.29, 95% CI: 0.18–0.37). The mediation effect in the model of T3 PTSD was more equally distributed among the three pain variables than in the model of T2 PTSD (Figure 1b).

#### MDE

Combat injury was associated with increased odds of past-30-day MDE at T2 (OR = 2.56, 95% CI: 1.74–3.76) compared to no combat injury. When T1 deployment stress and T2 pain variables were added to the model, pain interference (OR = 3.87, 95% CI: 2.58–5.81) and pain catastrophizing (OR = 1.74, 95% CI: 1.16–2.60) were associated with increased odds of MDE, but combat injury was no longer associated (Table 3; OR = 1.32, 95% CI: 0.81–2.15).

Combat injury also exhibited a significant association with past-30-day MDE at T3 (OR = 1.74, 95% CI: 1.07–2.85) that was eliminated when T2 pain interference (OR = 1.84, 95% CI: 1.35–2.52) and clinically significant pain intensity (OR = 1.80, 95% CI: 1.30–2.49) were included in the model (Table 3).

#### Joint mediation of the relation between combat injury and MDE by pain variables

Multiple mediation analysis (Figure 1c) revealed that service members with a combat injury had 1.91 times greater odds of past-30-day MDE at T2 than those without a combat injury (log odds = 0.65, 95% CI: 0.11–1.24). The joint mediation effect indicated 60% of the increase in odds of MDE due to combat injury was explained by the pain variables (log odds = 0.39, 95% CI: 0.26–0.52). Most of the mediation effect was attributable to pain interference (37.9%) and pain catastrophizing (19.9%). For T3 MDE, the total effect was not significant (log odds = 0.37, 95% CI: −0.19–0.79).

## Discussion

Results suggest that combat injury is associated with increased odds of new-onset pain interference, new-onset pain catastrophizing, PTSD, and MDE compared to no combat injury (irrespective of the presence of another painful condition) among combat-deployed US service members. In order to disambiguate the impact of combat injury broadly and pain specifically related to that injury, we assessed the cross-sectional relation of pain primarily attributed to a combat injury (i.e. combat injury–related pain) with pain experiences and mental health outcomes. We found that, compared to pain from other injuries and conditions, combat injury–related pain was associated with increased odds of PTSD and MDE, with the relation between combat injury–related pain and MDE potentiated by pain interference. This significant interaction of pain interference and combat injury–related pain on odds of MDE suggests that the functional impact of combat injury–related pain in particular may increase the odds of developing MDE. This finding is consistent with prior work demonstrating that the interference of pain with activities drives the association between pain and depression (Von Korff & Simon, 1996). Taken together with the elevated odds of PTSD among those with combat injury–related pain, this is evidence of a disproportionate mental health impact of pain from combat injuries versus other attributed causes, and suggests that the combination of chronic pain from a combat injury and pain-related functional impairment is associated with a particularly elevated risk of mood difficulties.

Evidence also indicates that pain-related experiences mediate the relation between combat injury and subsequent mental health difficulties. For example, pain catastrophizing and interference (but not intensity) explained significant proportions of the effect of combat injury on risk of *concurrent* PTSD and MDE (i.e. assessed at the same time point as the pain variables). This may reflect bidirectional effects of pain-related factors and mental health symptoms, whereby mental health symptoms magnify the psychological and functional burdens of pain and vice versa. Furthermore, all three pain-related factors mediated the relations between combat injury and PTSD, evaluated 6 months after the pain assessment. This suggests that the intensity of pain assessed several months after injury – along with its psychological and functional impacts – impacts the risk of chronic PTSD among those who sustain combat injury.

Findings are consistent with the theory that threat-based cues are relevant to both the initiation of chronic pain (i.e. threat of reinjury/increased pain) and the mutual-maintenance model of pain and mental health comorbidities (e.g. threat of reexperiencing trauma in PTSD; McAndrew et al., 2019; Sharp & Harvey, 2001). The significant mediation of the prospective relation between combat injury and PTSD by psychological factors of pain highlights the potentially mechanistic role of fears about pain in the relation between initial fearful experiences (i.e. combat injury) and long-term risk of PTSD. The unique contextual factors of military service may be particularly relevant to the role of threat-based cues and pain in the relation between injury and PTSD among service members and veterans. For example, training to remain vigilant could lead to a chronic sense of threat that could potentiate pain experiences and maintain PTSD. Furthermore, several psychological (e.g. suppression of emotions) and behavioral (e.g. lack of pacing of physical activity, pushing physical limits) predisposing factors for chronic pain are situationally adaptive in military culture. Taken together, findings support the supposition that aspects of military service could predispose chronic pain that could be precipitated by combat injury and perpetuated by psychological reactions to pain (e.g. pain catastrophizing). The study findings also broadly align with the chronic pain cycle, in which pain catastrophizing leads to less functional activity (e.g. work, social activities), which negatively impacts mood, social support, and physical fitness, which in turn leads to increased pain intensity and catastrophizing.

This study has limitations. We did not have a measure of injury severity or the type of injury experienced (e.g. penetrating, blast, etc.). While pain-related variables were collected more than 3 months following combat injury in both samples, indicating that any persisting pain interference or catastrophizing from the injury was consistent with a chronic pain process (i.e. pain lasting more than 3 months), future work including explicit measures of injury severity (e.g. the Injury Severity Score; Baker, ’Neill, Haddon, & Long, 1974) will better disambiguate the impact of injury severity versus pain sources and experiences. Furthermore, measures of combat injury and mental health outcomes were based on the service member’s report and not derived from a medical record or confirmed by a provider. Additionally, pain interference and catastrophizing were derived from a brief set of items. While these items are derived from validated measures and the internal consistencies of the items are satisfactory, further work examining the pain experiences of service members with more detailed assessments is warranted. Another limitation is the dichotomization of pain interference, pain catastrophizing, PTSD, and MDE, which artificially reduces these complex constructs into binary experiences. To mitigate this concern, the probable diagnoses of PTSD and MDE used in this study have been validated previously (e.g. Kessler et al., 2013a, 2013b). While they do not necessarily reflect the severity of symptoms, these carry the benefit of clinical relevance. Our results offer important insight into the impact of the presence of pain interference and pain catastrophizing on likely diagnosis of PTSD and MDE; however, future work would benefit from examining these constructs as dimensional variables to understand the impact of the severity of interference and catastrophizing on the severity of PTSD and MDE symptom burden. In particular, examination of the ways in which combat injury and pain factors affect each symptom cluster in PTSD (reexperiencing, avoidance, negative alterations of cognition and mood, and changes in arousal and reactivity) could lead to greater understanding of how combat injury and chronic pain mechanisms may drive comorbidity of chronic pain and PTSD.

Several treatment implications arise from these findings. First, results suggest that effective, early treatment of pain in service members may have benefits for both overall functioning and prevention of negative mental health outcomes. The early and comprehensive treatment of acute pain could prevent the transition to chronic pain through reduction in nociceptive distress, which can, in turn, reduce the burden of allodynia and hyperalgesia (i.e. increased pain sensitivity) following injury. Psychoeducation regarding risk factors and treatment for chronic pain delivered proactively to service members and reinforced after injury could also be beneficial. Second, traumatic sources of pain (e.g. combat injury) may respond to psychosocial pain treatments, particularly treatments that specifically target the mutual maintenance of PTSD and pain through fear processes (e.g. Emotion Awareness and Expression Therapy [EAET] for Chronic Pain; Yarns et al., 2024). Relatedly, the finding that attribution of pain to a combat injury is associated with pain-related functioning and mental health outcomes compared to other pain attributions suggests that treatments, such as EAET, that focus on reattributing pain from physical sources to mind/brain sources could also be effective (Ashar et al., 2023). Finally, the impact of pain interference and pain catastrophizing proximal to the injury on the development of later mental health conditions indicates that psychosocial treatments that target interference and catastrophizing (e.g. cognitive behavioral therapy for chronic pain) in the immediate aftermath of a combat injury may have broad-reaching benefits (Murphy et al., 2022).

Taken together, the results of this study suggest that both combat injury and the attribution of pain to a combat injury are closely, positively related to mechanisms of chronic pain – pain catastrophizing and interference – and may warrant enhanced screening and pain management interventions at the time of injury to mitigate the development of pain interference and pain catastrophizing, and subsequent chronic pain and mental health concerns. These findings are particularly relevant in the context of ongoing global conflicts, increasing the exposure of both civilians and military personnel to combat-related injury.

## Supporting information

10.1017/S0033291726103584.sm001 Wild et al. supplementary material Wild et al. supplementary material

## Supplementary material

The supplementary material for this article can be found at http://doi.org/10.1017/S0033291726103584.
