# Supplementary material for: Combat injury, pain, and mental health outcomes in US Army service members
Source: Psychol Med. 2026 Mar 24;56:e78. doi: 10.1017/S0033291726103584 (PMC13040298; doi:10.1017/S0033291726103584)
Supplement: Wild et al. supplementary material [file S0033291726103584sup001.docx]

**Supplementary Material**

To examine the impact of combat injury on the new onset of pain-related factors, we investigated the emergence of pain interference and pain catastrophizing in the 1058 soldiers who endorsed 0 out of 10 pain on the Numeric Rating Scale at the pre-deployment assessment (T0).

*New Onset Pain Interference*

Combat injury reported at T1 was associated with increased odds of new-onset pain interference at T2 (Supplementary Table 1; OR= 2.78, 95% CI: 1.97-3.93) in a logistic regression model that included other deployment stress, age, sex, race, BCT, and prior deployment history as covariates. These results were maintained for new onset of pain interference at T3 (Supplementary Table 2; OR= 1.55, 95% CI: 1.05-2.28).

*New Onset Pain Catastrophizing*

Combat injury reported at T1 was associated with increased odds of new-onset pain catastrophizing at T2 (Supplementary Table 1; OR= 2.75, 95% CI: 1.92-3.92) in a logistic regression model that included other deployment stress, age, sex, race, BCT, and prior deployment history as covariates. These results were maintained for new onset of pain catastrophizing at T3 (Supplementary Table 2; OR= 2.03, 95% CI: 1.24-3.30).

**Supplementary Table 1**

*Weights-Adjusted Logistic Regression of new onset pain interference and pain catastrophizing in PPDS T2*

| Effect | OR | 95% CI | | χ ^2^ | *p* |
| --- | --- | --- | --- | --- | --- |
|  |  | *LL* | *UL* |  |  |
| Pain Interference |  |  |  |  |  |
| Combat Injury | 2.78 | 1.97 | 3.93 | 33.72 | <0.0005 |
| Deployment Stress | 1.15 | 1.11 | 1.19 | 58.97 | <0.0005 |
| Age | 1.00 | 0.98 | 1.02 | 0.04 | 0.835 |
| Female | 1.08 | 0.66 | 1.75 | 0.09 | 0.759 |
| Non-Hispanic Black | 0.78 | 0.49 | 1.24 | - | - |
| Hispanic | 1.01 | 0.73 | 1.40 | - | - |
| Non-Hispanic Other | 1.07 | 0.73 | 1.57 | 1.57 | 0.667 |
| Previously Deployed Once | 0.98 | 0.76 | 1.27 | - | - |
| Previously Deployed 2+ | 0.80 | 0.58 | 1.10 | 2.13 | 0.344 |
| Pain Catastrophizing |  |  |  |  |  |
| Combat Injury | 2.75 | 1.92 | 3.92 | 30.77 | <0.0005 |
| Deployment Stress | 1.14 | 1.09 | 1.20 | 31.96 | <0.0005 |
| Age | 1.01 | 0.99 | 1.03 | 0.50 | 0.478 |
| Female | 1.16 | 0.72 | 1.86 | 0.36 | 0.547 |
| Non-Hispanic Black | 1.57 | 1.09 | 2.27 | - | - |
| Hispanic | 1.49 | 1.06 | 2.09 | - | - |
| Non-Hispanic Other | 1.10 | 0.63 | 1.91 | 8.07 | 0.045 |
| Previously Deployed Once | 1.07 | 0.79 | 1.46 | - | - |
| Previously Deployed 2+ | 0.94 | 0.65 | 1.34 | 0.64 | 0.727 |

*Note*. total *N* = 1058. OR = odds ratio; CI = confidence interval; *LL* = lower limit; *UL* = upper limit. Reference categories: Female: Male; Race: non-Hispanic White; Deployments: Not previously deployed. Models also adjusted for Brigade Combat Team.

**Supplementary Table 2**

*Weights-Adjusted Logistic Regression of new onset pain interference and pain catastrophizing in PPDS T3*

| Effect | OR | 95% CI | | χ ^2^ | *p* |
| --- | --- | --- | --- | --- | --- |
|  |  | *LL* | *UL* |  |  |
| Pain Interference |  |  |  |  |  |
| Combat Injury | 1.55 | 1.05 | 2.28 | 4.89 | 0.027 |
| Deployment Stress | 1.13 | 1.08 | 1.18 | 30.34 | <0.0005 |
| Age | 1.03 | 1.01 | 1.05 | 7.69 | 0.006 |
| Female | 1.51 | 0.76 | 2.99 | 1.37 | 0.242 |
| Non-Hispanic Black | 1.11 | 0.82 | 1.50 | - | - |
| Hispanic | 1.02 | 0.80 | 1.31 | - | - |
| Non-Hispanic Other | 0.71 | 0.46 | 1.09 | 3.16 | 0.367 |
| Previously Deployed Once | 0.98 | 0.75 | 1.27 | - | - |
| Previously Deployed 2+ | 0.79 | 0.57 | 1.09 | 2.18 | 0.336 |
| Pain Catastrophizing |  |  |  |  |  |
| Combat Injury | 2.03 | 1.24 | 3.30 | 8.02 | 0.005 |
| Deployment Stress | 1.12 | 1.03 | 1.21 | 7.79 | 0.005 |
| Age | 1.03 | 1.00 | 1.05 | 3.71 | 0.054 |
| Female | 1.15 | 0.64 | 2.09 | 0.22 | 0.640 |
| Non-Hispanic Black | 1.01 | 0.63 | 1.62 | - | - |
| Hispanic | 1.24 | 0.89 | 1.74 | - | - |
| Non-Hispanic Other | 1.29 | 0.73 | 2.28 | 2.00 | 0.573 |
| Previously Deployed Once | 0.78 | 0.57 | 1.05 | - | - |
| Previously Deployed 2+ | 0.81 | 0.56 | 1.17 | 3.08 | 0.215 |

*Note*. total *N* = 1058. OR = odds ratio; CI = confidence interval; *LL* = lower limit; *UL* = upper limit. Reference categories: Female: Male; Race: non-Hispanic White; Deployments: Not previously deployed. Models also adjusted for Brigade Combat Team.
